# Early Aggressive Immunotherapy Improves Functional Outcome in Chronic Immune Sensory Polyradiculopathy

**DOI:** 10.1155/2020/6595086

**Published:** 2020-02-20

**Authors:** Jasmine Shimin Koh, James Wei Min Tung, Genevieve Lynn Yu Tan-Yu, Thirugnanam Umapathi

**Affiliations:** ^1^Department of Neurology, National Neuroscience Institute, 11 Jalan Tan Tock Seng, Singapore 308433; ^2^Lee Kong Chian School of Medicine, Nanyang Technological University, 59 Nanyang Dr., Experimental Medicine Building, Singapore 636921

## Abstract

Chronic immune sensory polyradiculopathy (CISP) is an uncommon and treatable inflammatory disorder of the proximal sensory nerve roots. Patients typically present with severe sensory ataxia, normal motor examination, unsteady gait, and normal nerve conduction studies (NCS). We describe an elderly man who presented with a two-week history of progressive numbness of both legs and recurrent falls. He had hyporeflexia, normal strength, severe proprioceptive, and vibration sense loss in both lower limbs and was unable to stand or walk because of severe sensory ataxia. The NCS and MR scan of the spine were normal. Tibial somatosensory evoked potentials revealed proximal conduction defect and localized the pathology to the lumbar sensory nerve roots proximal to the dorsal root ganglion. Cerebrospinal fluid showed cytoalbuminergic dissociation suggestive of inflammation. CISP was diagnosed; he was given aggressive immunotherapy consisting sequentially of corticosteroids with mycophenolate mofetil and three cycles of intravenous immunoglobulin after which he regained independent mobility. Unlike previous reports where patients presented months-years after symptom onset and improved after single-line immunotherapy, our patient presented fairly acutely and made dramatic improvement only after aggressive combination therapy. We urge physicians to recognize this uncommon neurologic cause of sensory ataxia where early aggressive treatment is crucial for better functional outcomes.

## 1. Introduction

First described by Sinnreich et al. in 2004 [1], chronic immune sensory polyradiculopathy (CISP) is an uncommon and probably underrecognized cause of peripheral sensory ataxia affecting the sensory nerve roots proximal to the dorsal root ganglion (DRG). Though some patients may have concomitant involvement of the proximal motor nerve roots and are termed chronic immune sensorimotor polyradiculopathy (CISMP) [2–5], others have disease strictly confined to the proximal sensory nerve roots. Publications related to CISP [6–8] remain rare and limited since the entity was first described. Here, we describe a case report of an elderly man who was diagnosed with CISP and improved remarkably only after several courses of combination immunotherapy, emphasizing the importance of prompt recognition and aggressive treatment.

## 2. Case Presentation

In this study, we describe an 80-year-old man presented with progressive feet numbness, unsteady gait, and recurrent falls for two weeks. He did not have limb weakness and bladder or bowel disturbance. He had severely impaired lower limb position and vibration sense as well as decreased deep tendon reflexes. Strength was normal, and Babinski's sign was absent. Cerebellar and cranial nerve examinations were normal. He was unable to stand or walk due to severe sensory ataxia. Initial differential diagnoses were sensory neuronopathy, CIDP, and dorsal spinal column pathology. Nerve conduction study (NCS), syphilis and HIV serologies, serum neuronal antibodies (anti-Hu, anti-Yo, anti-Ri, anti-CRMP5, antiamphiphysin, anti-Ma, anti-Ta, anti-SOX-1, and anti-GAD65), extractable nuclear antigen antibodies, vitamin B12, folate, and copper levels were normal. A nonenhanced MR scan of the spine showed normal spinal cord structure and signal and chronic lumbar degenerative changes with osteophyte and disc bulges. This did not explain the patient's relatively acute sensory ataxia. There was no nerve root thickening, enlargement, or enhancement seen possibly related to not administering contrast for the scan. CT brain showed normal brain parenchyma and cerebellum. Somatosensory evoked potentials (SSEP) and lumbar puncture were subsequently performed. Tibial SSEP (Table 1) revealed absent responses at lumbar, subcortical, and cortical points, suggesting proximal conduction defect and localizing the pathology to the lumbar sensory nerve roots proximal to the dorsal root ganglion. Median SSEP was normal at Erb's, cervicomedullary, and cortical points. Cerebrospinal fluid (CSF) showed cytoalbuminergic dissociation (nucleated cell 2 cells/*µ*L (reference range 0–5 cells/*µ*L) and protein 0.93 g/L (reference range 0.10–0.40 g/L)), indicative of inflammation. Ganglioside antibodies such as GD1b seen in acute and chronic sensory neuropathy with ataxia were not available. The constellation of clinical features, normal NCS, characteristic SSEP abnormalities, and raised CSF protein prompted a diagnosis of CISP.

Four days after admission, he was given 1 g of intravenous methylprednisolone daily for five days followed by oral prednisolone 1 mg/kg. He did not improve; hence, intravenous immunoglobulins (IVIg) were added at a dose of 0.4 g/kg daily for five days. Mycophenolate mofetil was also started as a steroid-sparing agent while the prednisolone dose was slowly tapered. He made minimal improvement over several weeks and remained functionally impaired. He was treated with another two courses of monthly IVIg before significant improvement in function was observed. Modified Rankin scale (mRS) improved from 4 to 1, while Berg balance score improved from 31/56 to 44/56 (Table 2). A repeat NCS remained normal (Table 3) while tibial SSEP suggested improvement with nonlocalized slowing (Table 1). Four months after the last IVIg course, he was able to walk independently. He is maintained on mycophenolate mofetil and has been in remission since.

## 3. Discussion

CISP is an immune-mediated inflammation of the proximal sensory nerve roots characterized by peripheral sensory ataxia, normal motor examination, normal motor and sensory NCS, characteristic SSEP abnormalities, raised CSF protein on spinal tap, and thickened nerve roots on MR imaging [1].

Initial symptoms may be asymmetric and start with paresthesia, numbness, or pain of the distal lower limbs. These symptoms gradually worsen and spread proximally to the hips and upper limbs with more symmetric involvement. Duration from symptom onset to medical consult ranges from few months to 18 years [1,6–8]. Neurologic examination indicates large-fibre sensory loss and sensory gait ataxia. Muscle strength is often preserved except in CISMP where there is concomitant motor nerve root involvement [2–5].

Histologic findings of three cases from the largest series [1] diagnosed with CISP that underwent lumbar sensory rootlet biopsy showed presence of endoneurial macrophages, lack of degenerating profiles, and features of chronic demyelination and remyelination with numerous onion-bulb formations. These features support primary demyelination of proximal sensory nerve roots, similar to changes observed in the peripheral nerves of patients with CIDP suggesting that CISP may be an additional phenotype along the CIDP spectrum [8–10]. However, unlike CIDP, the distal nerve segments are spared in CISP, as evidenced by normal NCS. Characteristic SSEP abnormalities include absent or delayed proximal lumbar/cervical responses, while the contrast-enhanced MR scan of the spine could show thickened enhancing nerve roots. Spinal tap typically shows cytoalbuminergic dissociation [1, 6–8].

Through this case, we would like to raise awareness of this treatable immune-mediated condition among physicians and caution against wrongly attributing the sensory ataxia and unsteady gait to a functional gait disorder especially when routine NCS and spine imaging are normal. Prompt recognition in addition to specific evaluation with SSEP, spinal tap, and contrast-enhanced MR scan of the spinal nerve roots will help clinch the diagnosis.

In addition, we emphasize the importance of early and aggressive immunotherapy. The immunopathology in CISP is at the central processes of the spinal sensory nerve roots, proximal to the dorsal root ganglion. Nerve regeneration here is likely to be less robust because of the need to form connections with the central nervous system [11], possibly inhibited by factors such as Nogo-A [12]. Other pathologies at this site, e.g., traumatic nerve root avulsion, likewise have poor regenerative prognosis. Although there are currently no randomized trials to guide treatment in CISP owing to its rarity, we believe the importance of aggressive and early immunotherapy in CISP before Wallerian degeneration is advanced in the spinal sensory nerve roots. Our patient presented early compared with other cases in the literature [1,6,7]. This and the use of combination immunotherapy which has not been described before in CISP, we believe, led to his good and complete recovery after seven months of treatment.

Finally, we suggest incorporating follow-up SSEP in addition to clinical functional scores (mRS and Berg balance scale) to gauge treatment response. Our experience corroborates with a previous report that SSEP may serve as an additional aid for modulation of immunotherapy [7].

## 4. Conclusion

CISP is a rare and treatable cause of peripheral sensory ataxia. Prompt recognition and early institution of aggressive combination immunotherapy improve functional outcomes.

## Figures and Tables

**Table 1:** Initial and repeat tibial somatosensory evoked potentials.

	Initial		Repeat (four months after treatment)		
Waveform	Left (ms)	Right (ms)	Left (ms)	Right (ms)	Normal reference
Popliteal fossa	8.40	8.90	9.1	9.1	—
Lumbar point (N21)	Absent	Absent	Absent	Absent	<22.0
Subcortical (P31)	Absent	Absent	32.3	34.5	<31.6
Cortical (P37)	Absent	Absent	Absent	Absent	<40.2

**Table 2:** Functional scores in relation to time course and treatment.

	On admission	1.5 weeks	5 weeks	2.5 months	4 months	7 months
Immunosuppressant	Corticosteroids and mycophenolate mofetil					
Intravenous immunoglobulins (IVIg)	—	1^st^ IVIg	2^nd^ IVIg	3rd IVIg	—	—
Modified Rankin scale (mRS)	4	4	4	4	1	0
Berg balance score	Not assessed	31/56	34/56	39/56	44/56	Not assessed

**Table 3:** Initial and repeat (performed 4 months apart) nerve conduction study parameters.

Sensory nerve conduction						
	Right median nerve (antidromic)			Right ulnar nerve (antidromic)		
	Initial	Repeat	Normal limit∗	Initial	Repeat	Normal limit^*∗*^
Peak latency (ms)	3.6	3.2	<3.9	3.0	3.0	<3.0
Amplitude (*µ*V)	35	26	>14	20	23	>9.0
Velocity (m/s)	50	55	>50	50	51	>50
	Right sural (antidromic)					
	Initial	Repeat	Normal limit∗			
Peak latency (ms)	3.8	3.7	<4.5			
Amplitude (*µ*V)	7	8	>7.0			
Velocity (m/s)	48	49	>40			

Motor nerve conduction^						

	Right median nerve (antidromic)			Right ulnar nerve (antidromic)		
	Initial	Repeat	Normal limit∗	Initial	Repeat	Normal limit∗
Distal latency (ms)	3.7	—	<4.5	2.9	—	<3.0
Amplitudes (mV) (distal/proximal)	7.7/6.6^#^	—	>5.5	11.1/9.5^#^	—	>7.0
Velocity (m/s)	50	—	>50	56	—	>50
F-wave latency (ms)	26.2	—	22–32	27.4	—	21–29
	Right tibial nerve (antidromic)					
	Initial	Repeat	Normal limit∗			
Distal latency (ms)	4.6	—	<4.6			
Amplitudes (mV) (distal/proximal)	8.7/7.9^#^	—	>5.0			
Velocity (m/s)	40	—	>42			
F-wave latency (ms)	47.8	—	38–52			

∗Normative data derived from 245 age-height matched controls; ^#^measurements following stimulation at the wrist/antecubital fossa for median nerve, at the wrist/above elbow for ulnar nerve, and at the ankle/popliteal fossa for tibial nerve; ^repeat motor nerve conduction study was not performed as the patient had no motor deficits.
